# Thalidomide Inhibits TGF-β1-induced Epithelial to Mesenchymal Transition in Alveolar Epithelial Cells via Smad-Dependent and Smad-Independent Signaling Pathways

**DOI:** 10.1038/s41598-017-15239-2

**Published:** 2017-11-07

**Authors:** Xian-Long Zhou, Peng Xu, Hai-Hua Chen, Yan Zhao, Jun Shen, Cheng Jiang, Shan Jiang, Shao-Zhou Ni, Bing Xu, Lei Li

**Affiliations:** Emergency Center, Zhongnan Hospital of Wuhan University, 169 Donghu Road, Wuchang, Wuhan, Hubei 430071 P. R. China

## Abstract

Recent evidence indicates that the epithelial to mesenchymal transition (EMT) in primary alveolar cells (AECs) plays an important role in idiopathic pulmonary fibrosis (IPF). *In vivo* models have suggested that thalidomide (THL) has anti-fibrotic effects against pulmonary fibrosis, but the underlying mechanism of this effect is not clear. This study investigated whether THL regulates alveolar EMT and the possible mechanisms underlying this process. CCL-149 cells were treated with TGF-β1 in the presence of THL at the indicated concentrations. EMT was assessed by changes in cell morphology and in phenotypic markers. Signaling pathways involved in EMT were characterized by western blot analysis. THL inhibited the TGF-β1 induction of α-SMA, vimentin, MMP-2/-9 and collagen type IV expression and restored the morphological changes in primary alveolar epithelial cells caused by TGF-β1. TGF-β1 induction of α-SMA expression was partially dependent on the activation of p38, JNK, ERK, Akt, Smad 2 and Smad3. Moreover, THL inhibited TGF-β1-induced phosphorylation of p38, JNK, ERK, Akt, GSK3β, Smad 2 and Smad3 without altering the total expression levels of those proteins. These findings indicate that TGF-β1-induced EMT in alveolar epithelial cells is inhibited by THL via both Smad-dependent and non-Smad-dependent signaling pathways and suggests therapeutic approaches for targeting this process in pulmonary fibrosis.

## Introduction

Recent evidence indicates that the epithelial to mesenchymal transition (EMT) plays an important role in the development of pulmonary fibrosis^1^. Several mediators, including cytokines and growth factors, are thought to induce EMT, and TGF-β has been shown to be the major inducer of EMT in carcinogenesis and fibrosis^2^. A previous study reported that alveolar epithelial cells (AECs) undergo EMT when exposed to TGF-β1, indicating that AECs may serve as a novel source of myofibroblasts in pulmonary fibrosis^3^. Following exposure to TGF-β1, AECs undergo EMT as evidenced by decreased expression of epithelial markers (AQP5, ZO-1 and E-Cadherin), increased expression of mesenchymal markers (α-SMA, vimentin and desmin) and a transition to a fibroblast-like morphology^4^. In addition, fibrosis- or TGF-β1-induced EMT is mainly mediated by Smad-dependent (Smad2/3) and Smad-independent pathways (MAPK, PI3 kinase, Ras and Wnt signaling pathways, etc.)^5^. Thus, researchers are investigating the possibility that the regulation of Smad-independent and Smad-dependent pathways in AECs could result in reduced fibrogenesis through the attenuation of TGF-β1-induced EMT^6,7^. Although the role of EMT in organ fibrosis has recently been questioned by lineage-tracing studies^8,9^, experimental evidence suggests an important role of EMT in the pathogenesis of pulmonary fibrosis^10,11^.

Thalidomide (THL) has been recognized as a therapeutic agent for multiple myeloma (MM). Recent studies have shown that thalidomide has anti-fibrotic effects in animal models of bleomycin-induced^12^ and paraquat-induced pulmonary fibrosis^13^. Although the anti-fibrotic effects of thalidomide in pulmonary fibrosis have been demonstrated, the exact mechanism of this effect remains poorly understood. The results of previous studies have suggested that the anti-fibrotic effects of thalidomide on pulmonary fibrosis might be related to suppression of the ERK1/2 signaling pathway^14^. *Liu et al*. reported that thalidomide is able to inhibit JNK signaling in an animal model of pulmonary fibrosis^15^. In addition, a study by *Liang et al*. suggested that thalidomide has an anti-fibrotic effect that is caused by suppression of TGF-β1-induced p38 and Smad3 signaling^16^. Considering the anti-fibrotic effect of thalidomide in pulmonary fibrosis, the possible mechanisms of that effect and the characteristics of EMT in pulmonary fibrosis, we hypothesized that thalidomide inhibits TGF-β1-induced EMT in AECs. In the present study, we induced EMT *in vitro* to investigate the potential effects of thalidomide on alveolar EMT and the possible mechanisms of this effect.

## Results

### Thalidomide reversed TGF-β1-induced EMT in AECs

**Figure 1:**
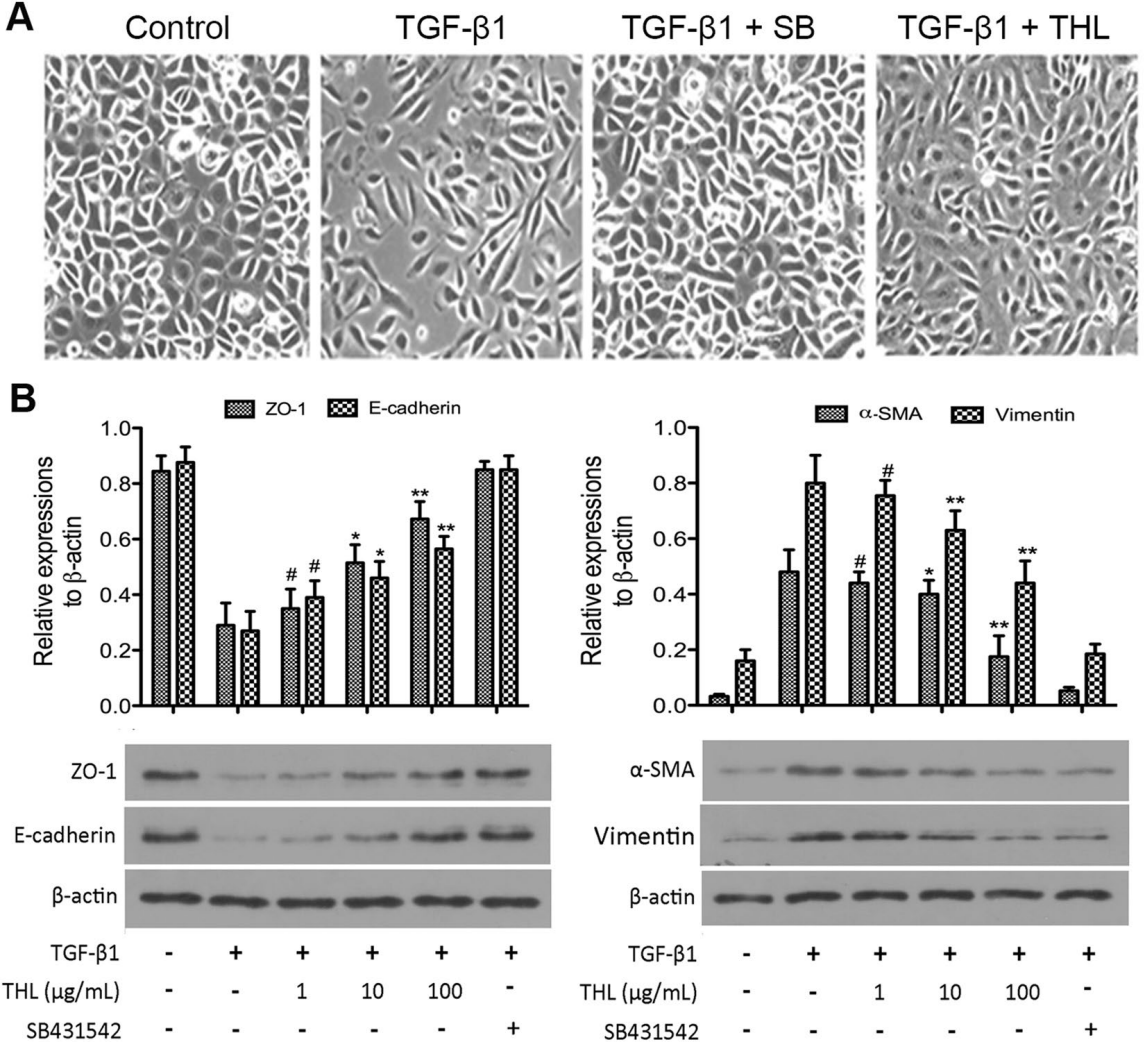
Thalidomide (THL) attenuates TGF-β1-induced EMT in CCL-149 cells. (**A**) Morphological changes (200×) in CCL-149 cells treated with vehicle or TGF-β1 (5 ng/mL) for 48 hours in the presence of vehicle (DMSO), SB431542 (5 μM), or THL (100 μg/mL). (**B**) CCL-149 cells stimulated with TGF-β1 (5 ng/mL) were treated with THL (1, 10, or 100 μg/mL) or SB431542 (5 μM) for 48 hours, and the lysates were analyzed for ZO-1, E-Cadherin, α-SMA and vimentin. β-Actin was used as the loading control. Data are expressed as the means ± SD, and n = 3 in each group. THL: thalidomide; SB: SB431542, TGF-β1R-Kinase inhibitor; ZO-1: zonula occludens-1; α-SMA: α-smooth muscle actin; ^#^p < 0.05, ^##^p < 0.01 compared to the TGF-β1 group (untreated); ^*^p < 0.05, ^**^p < 0.01 compared to all other groups.

**Figure 2:**
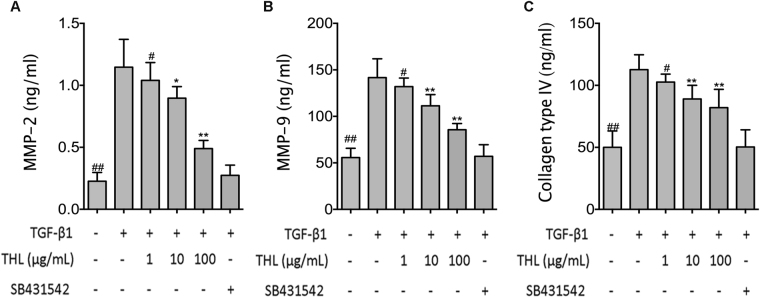
ELISA analysis. CCL-149 cells stimulated with TGF-β1 (5 ng/mL) were treated with THL (1, 10, or 100 μg/mL) or SB431542 (5 μM) for 48 hours, and then the cell culture media were collected for ELISA analysis of MMP-2 (**A**), MMP-9 (**B**) and collagen type IV (**C**) secretions. THL: thalidomide; SB431542: TGF-β1R-Kinase inhibitor; MMP-2, and -9: matrix metalloproteinase-2, and -9; ^#^p < 0.05, ^##^p < 0.01 compared to the TGF-β1 group (untreated); *p < 0.05, **p < 0.01 compared to all other groups.

**Figure 3:**
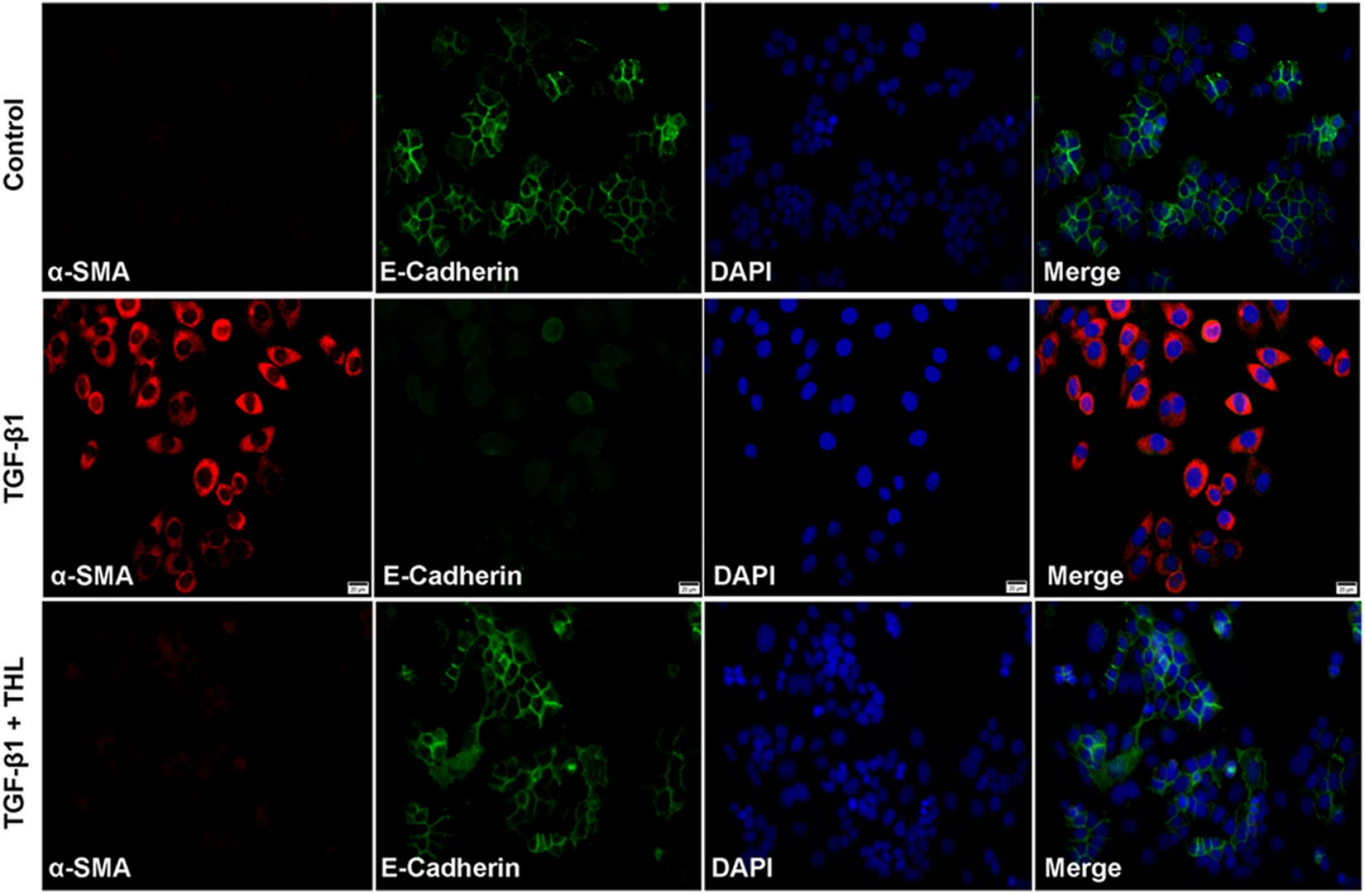
Thalidomide (THL) reverts TGF-β1-induced EMT as detected by confocal immunofluorescence microscopy. CCL-149 cells were grown on coverslips and treated with TGF-β1 in the presence or absence of THL (100 μg/mL) for 48 h. Cells were fixed and stained with α-SMA and E-Cadherin antibodies followed by incubation with fluorescently-tagged secondary antibodies. DAPI was used to stain nuclei. α-SMA (red), E-Cadherin (green), and nuclei (blue) were visualized by confocal fluorescence microscopy (400×).

Stimulation with 5 ng/mL TGF-β1 for 48 h resulted in apparent changes in cellular morphology and changes in the expression of cellular markers. As seen in Fig. 1A, treatment with thalidomide restored the morphological changes observed in cells after TGF-β1 stimulation. The majority of TGF-β1-treated cells in the thalidomide-treated groups showed an epithelial morphology instead of a fibroblast-like morphology. Western-blot assays showed that thalidomide significantly inhibited the TGF-β1-induced increases in α-SMA and vimentin in a dose-dependent manner (p < 0.05 for both α-SMA and vimentin). Treatment with thalidomide significantly prevented the TGF-β1-induced decrease in the epithelial markers ZO-1 and E-Cadherin (p < 0.05 for both ZO-1 and E-Cadherin) (Fig. 1B). In addition, ELISAs showed that pretreatment with TGF-β1 significantly increased the secretion of MMP-2, MMP-9 and collagen type IV in CCL-149 cells. However, thalidomide abrogated these changes in a dose-dependent manner (p < 0.05) (Fig. 2). Moreover, immunofluorescence microscopy demonstrated localized expression of the epithelial marker E-Cadherin and the mesenchymal marker α-SMA in CCL-149 cells (Fig. 3). Cells in the control group expressed significant amounts of E-Cadherin and low levels of α-SMA on their cell membranes. TGF-β1 markedly reduced the expression of E-Cadherin and increased the expression of α-SMA. However, cells concurrently treated with TGF-β1 and thalidomide retained high levels of expression of the epithelial marker E-Cadherin without a significant increase in the expression of the mesenchymal marker α-SMA.

### Thalidomide inhibited TGF-β1-induced phosphorylation of p38, JNK and ERK

**Figure 4:**
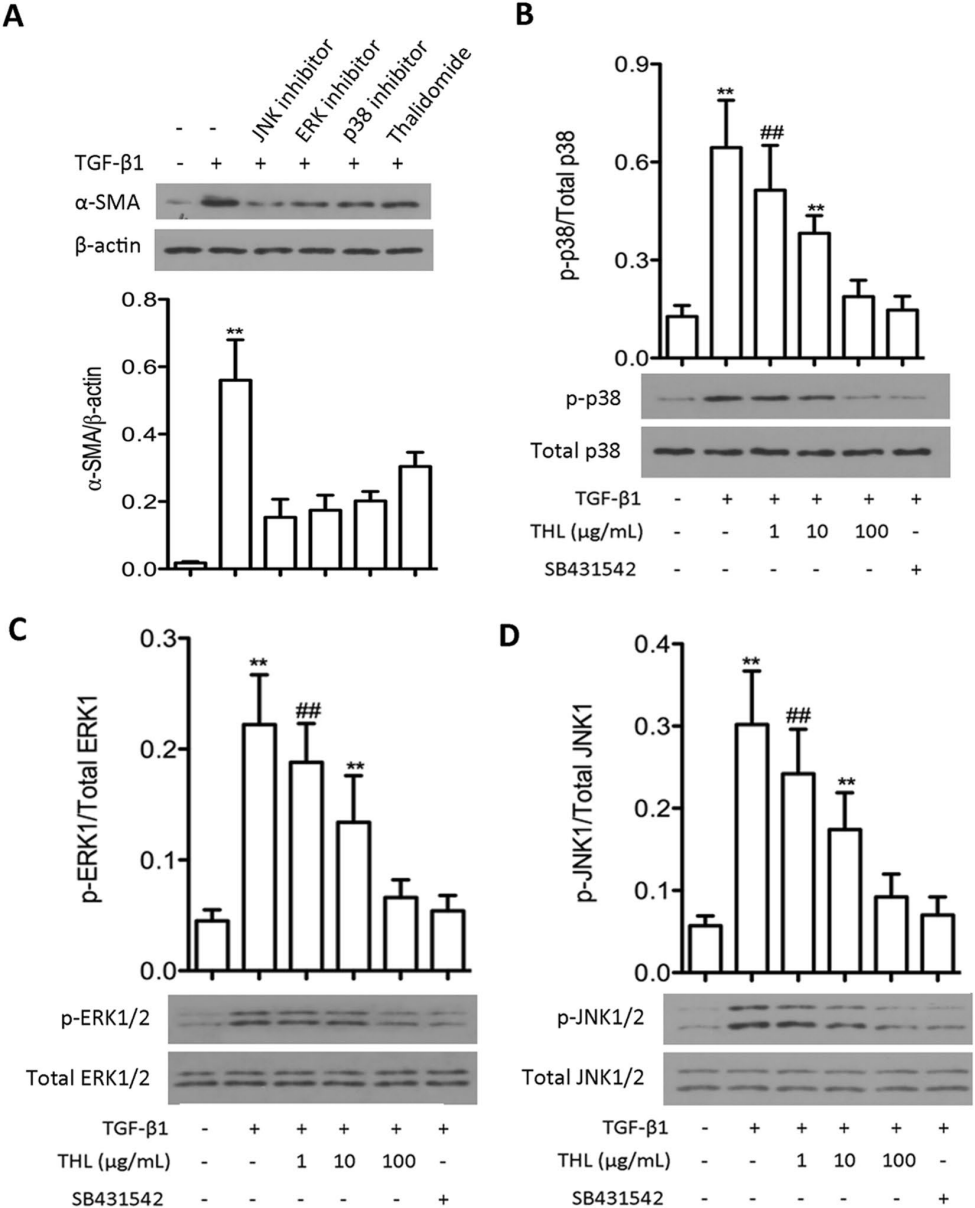
Thalidomide (THL) inhibits TGF-β1-mediated phosphorylation of MAPK in CCL-149 cells. (**A**) Cells were preincubated for 1 h with JNK inhibitor SP600125 (10 μM), ERK inhibitor PD098059 (40 μM), and p38 inhibitor SB203580 (20 μM) and then stimulated with TGF-β1 (5 ng/mL) for 48 h. Then, cells were collected for α-SMA measurement by western blot. (**B**,**C** and **D**) Following TGF-β1 treatment, JNK, ERK and p38 were markedly phosphorylated in CCL-149 cells. Treatment with THL (1, 10, or 100 μg/mL) or SB431542 (5 μM) attenuated JNK, ERK and p38 phosphorylation. β-Actin was used as a loading control. Data are expressed as the means ± SD, and n = 3 in each group. THL: thalidomide; SB431542: TGF-β1R-Kinase inhibitor; α-SMA: α-smooth muscle actin; ^##^p < 0.01 compared to the TGF-β1 group (untreated); ^**^p < 0.01 compared to all other groups.

TGF-β1-induced EMT is associated with the activation of numerous signaling pathways. It is well known that the MAPKs, including p38, JNK, and ERK, can be activated by TGF-β1 in pulmonary epithelial cells. In this study, we first confirmed the role of p38, JNK, and ERK in TGF-β1-induced EMT. When cells were treated with inhibitors specific for these proteins, including SB203580 (p38 inhibitor), PD098059 (JNK inhibitor) and SP600125 (ERK inhibitor), the activation of p38, JNK and ERK by TGF-β1 and the expression of the mesenchymal marker α-SMA was significantly inhibited (p < 0.05) (Fig. 4A). Importantly, we observed that thalidomide inhibited the activation of these MAPKs after TGF-β1 treatment in a dose-dependent manner (p < 0.05) without altering the total expression levels of those proteins (Fig. 4B). These results indicate that thalidomide might inhibit TGF-β1-induced EMT in pulmonary epithelial cells through the inhibition of MAPK signaling pathways.

### Thalidomide inhibited TGF-β1-induced phosphorylation of Akt and GSK-3β

**Figure 5:**
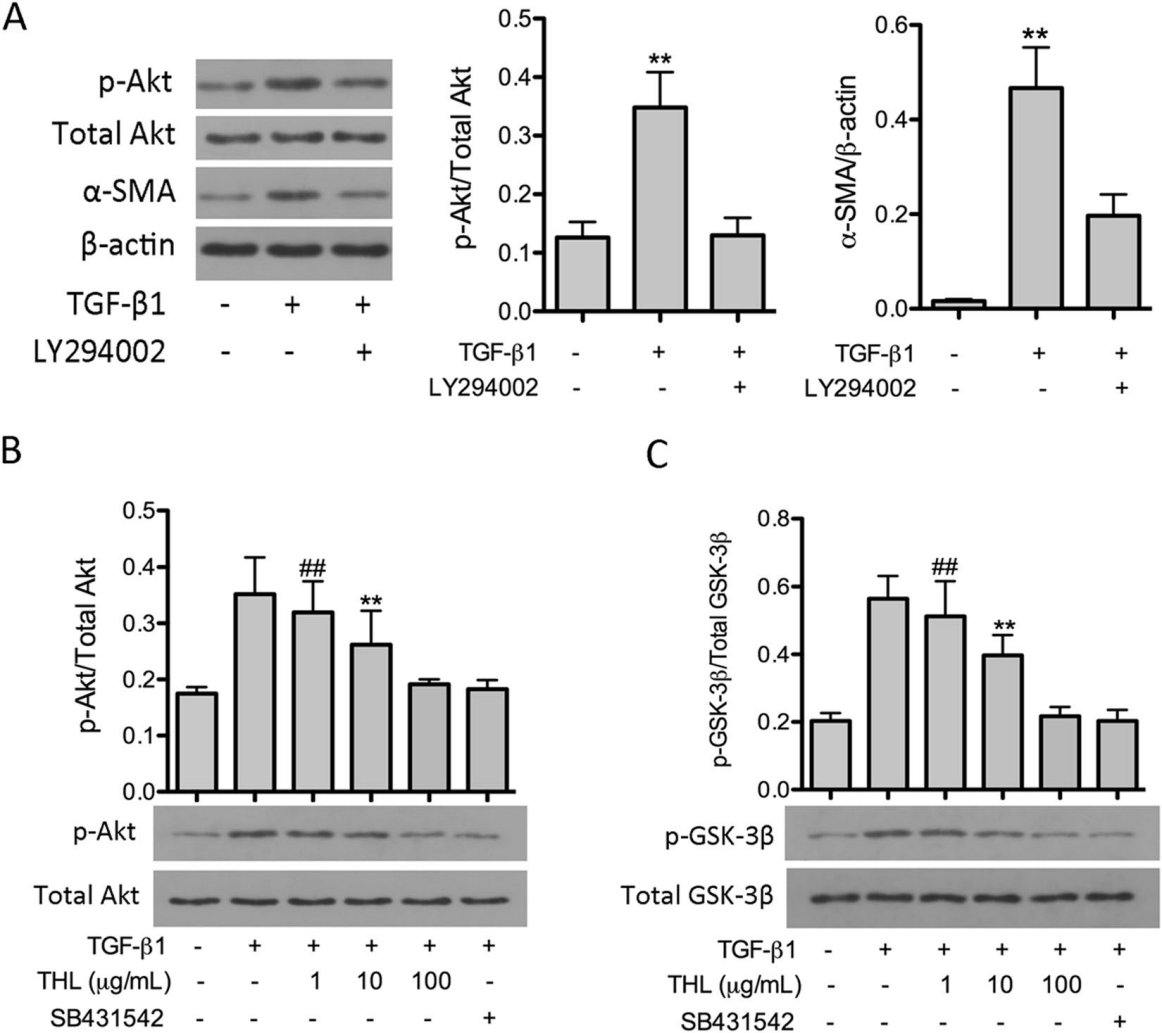
Thalidomide (THL) inhibits TGF-β1-mediated phosphorylation of Akt and GSK-3β in CCL-149 cells. (**A**) Concomitant treatment with PI3K/Akt inhibitor LY294002 (3 μM) and TGF-β1 (5 ng/mL) suppressed Akt phosphorylation and α-SMA expression in CCL-149 cells. (**B** and **C**) Following TGF-β1 treatment, Akt and GSK-3β were markedly phosphorylated in CCL-149 cells. Treatment with THL (1, 10, or 100 μg/mL) or SB431542 (5 μM) attenuated phosphorylation of both Akt and GSK-3β. β-Actin was used as the loading control. Data are expressed as the means ± SD, and n = 3 in each group. THL: thalidomide; SB431542: TGF-β1R-Kinase inhibitor; α-SMA: α-smooth muscle actin; ^##^p < 0.01 compared to the TGF-β1 group (untreated); ^**^p < 0.01 compared to all other groups.

A previous study confirmed that TGF-β1 induces the phosphorylation of Akt and its downstream protein GSK-3β in primary AECs^17^. In this study, we also found that TGF-β1 induced phosphorylation of Akt in CCL-149 cells *in vitro*. In addition, treatment with the PI3-K/Akt specific inhibitor LY294002 significantly inhibited the TGF-β1-induced phosphorylation of Akt and the subsequent induction of α-SMA expression (Fig. 5A). Furthermore, TGF-β1 treatment also induced the phosphorylation of GSK-3β in AECs. These results suggest that the Akt/GSK-3β pathway is involved in TGF-β1-induced EMT in CCL-149 cells. However, treatment with thalidomide inhibited TGF-β1-induced Akt and GSK-3β phosphorylation (Fig. 5B and C) in a dose-dependent manner (p < 0.05). In addition, as shown above, thalidomide also inhibited TGF-β1-induced production of α-SMA and vimentin and restored the morphological changes caused by TGF-β1. Thus, we conclude that the Akt/GSK-3β pathway is involved in thalidomide-mediated anti-EMT effects.

### Thalidomide inhibited TGF-β1-induced phosphorylation of Smad 2/3

**Figure 6:**
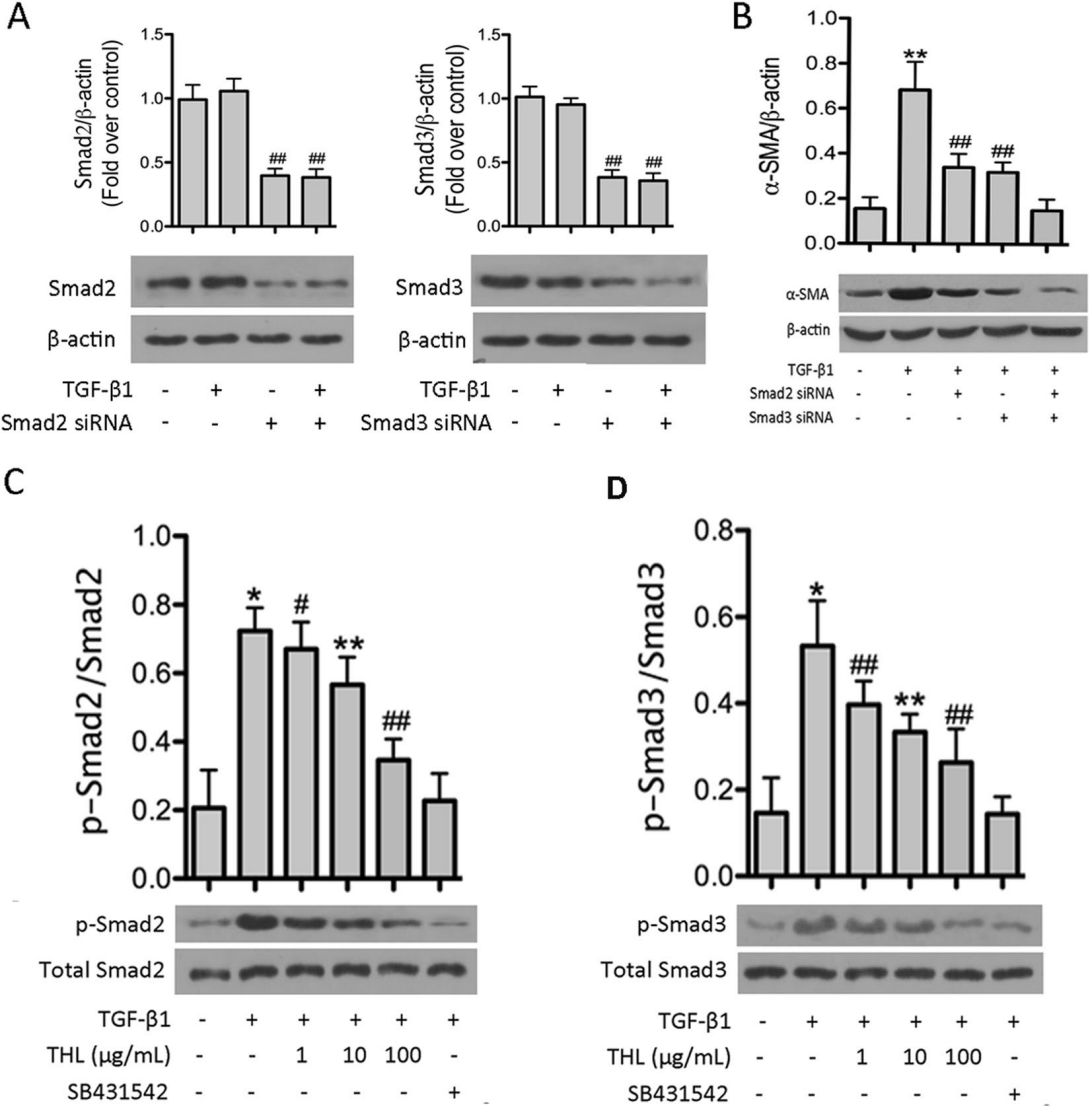
Thalidomide (THL) inhibits TGF-β1-mediated phosphorylation of Smad2 and Smad3 in CCL-149 cells. (**A**) CCL-149 cells were transfected with Smad2 and Smad3 siRNAs. Smad2 and Smad3 siRNA treatment resulted in approximately a 60% decrease in the density of Smad protein bands 48 hours after transfection. (**B**) Cells transfected with Smad2 siRNA and/or Smad3 siRNA were treated with or without TGF-β1 (5 ng/mL) for 48 h. Cells were lysed and α-SMA levels were evaluated by western blot. (**C**) Cells were stimulated with TGF-β1 (5 ng/mL) in the presence of vehicle, SB431542 (5 μM) or increasing doses of THL (1, 10, and 100 μg/mL) for 48 h. Then, cells were lysed and the phosphorylation of Smad2 and Smad3 was evaluated by western blot. β-Actin was used as a loading control. Data are expressed as the means ± SD, and n = 3 in each group. THL: thalidomide; SB431542: TGF-β1R-Kinase inhibitor; α-SMA: α-smooth muscle actin; ^#^p < 0.05, ^##^p < 0.01 compared to the TGF-β1 group (untreated); ^*^p < 0.05, ^**^p < 0.01 compared to all other groups.

Smad transcription factors are well-known mediators of TGF-β1 signaling. A previous study showed that Smads are activated by TGF-β1 and regulate EMT-related changes in pulmonary epithelial cells^18^. To confirm the role of Smad2/3 in TGF-β1-induced EMT, Smad2 and Smad3 were knocked down using siRNAs. As seen in Fig. 6A, the knockdown of Smad2 and Smad3 proteins was comparable in cells with or without TGF-β1 treatment. We also investigated whether co-treatment with Smad2 and Smad3 siRNAs enhanced the knockdown of target genes in this study. However, no significant changes were observed in cells co-treated with Smad2/3 siRNAs compared to cells treated with single siRNAs (data not shown). Moreover, we investigated the effects of the knockdown of Smad2 and Smad3 on TGF-β1-induced α-SMA expression. The TGF-β1-induced increase in α-SMA expression was significantly attenuated by knockdown of both Smad2 and Smad3. Furthermore, the TGF-β1-induced increase in α-SMA expression was completely inhibited in cells transfected with both Smad2 and Smad3 siRNAs (Fig. 6B). These results confirm that both Smad2 and Smad3 are involved in the TGF-β1-induced increase in α-SMA expression in CCL-149 cells. Importantly, cells concurrently treated with TGF-β1 and thalidomide showed significantly lower levels of α-SMA expression and decreased phosphorylation of Smad2/3 compared to cells treated with TGF-β1 in the absence of thalidomide (Fig. 5C). These results indicate that the inhibitory effect of thalidomide on TGF-β1-induced EMT is associated with the activation of both Smad2 and Smad3.

## Discussion

This study is the first to demonstrate that thalidomide inhibits TGF-β1-induced alveolar epithelial to mesenchymal transition (EMT) in CCL-149 cells. Thalidomide significantly reduced the expression of mesenchymal markers, increased the expression of epithelial markers after TGF-β1 stimulation, and reverted the TGF-β1-induced morphological changes in alveolar epithelial cells. We also found that the inhibitory effects of thalidomide on TGF-β1-induced EMT may be related to inhibition of the TGF-β1-mediated signaling pathways Smad2/3, Akt/GSK-3β and MAPK.

Currently, increasing attention has been given to the process of EMT and its possible role in the pathogenesis of pulmonary fibrosis. Numerous studies have reported EMT as a response to TGF-β1 treatment *in vitro* in alveolar epithelial cells^19,20^. The mechanism of TGF-β1-induced EMT is complicated because TGF-β1 signals are transmitted through multiple pathways, including the Smad, MAPK and PI3K pathways. The TGF-β1 signal itself is predominantly transduced by the Smad proteins, including Smad2, Smad3 and Smad4^21^. Binding of TGF-β1 to its receptors results in the phosphorylation of Smad proteins, causing translocation of the Smad complex into the nucleus where it regulates the expression of target genes. Although both Smad2 and Smad3 are important for the regulation of TGF-β1 signals, the mechanisms of action and the functions of these two Smads are different. Experimental Smad3-deficient mouse models of EMT and fibrosis demonstrated that Smad3 signaling is important in fibrosis^22^; however, the role of Smad2 in TGF-β1-induced EMT remains unclear. In the present study, we observed that the TGF-β1-induced phosphorylation of Smad2 and Smad3 and the TGF-β1-induced increase in α-SMA production were partially dependent on Smad2 and Smad3 and were reversed by knockdown of both Smad2 and Smad3. These results are consistent with those of previous reports^23^. Moreover, the results of our study suggest that thalidomide inhibits TGF-β1-induced EMT in pulmonary epithelial cells by blocking the phosphorylation of both Smad2 and Smad3. However, previous studies demonstrated that Smad3, but not Smad2, is required for the fibrotic response to TGF-β1^24,25^. In addition, a study of alterations in gene expression during EMT in IHAEo^−^ cells showed that only collagen I gene expression was Smad2/3 dependent; all other alterations in gene expression during EMT appeared to be Smad independent^18^. The discrepancies between the findings of these studies may be due to the cell types used. However, the Smad dependency of EMT changes in CCL-149 cells (other than those studied here) requires further investigation.

In addition to the Smad pathways, TGF-β utilizes several non-Smad pathways, such as the MAP kinase and phosphatidylinositol-3-kinase/AKT pathways, to regulate a wide array of cellular functions. To date, the best-characterized non-Smad pathway utilized by TGF-β is the p38 MAPK and JNK signaling cascade. It is well known that TGF-β can rapidly activate p38 MAPK and JNK through MKK3/6^26^ and MKK4^27^, respectively. In addition, the p38 MAPK and JNK pathways play very important roles in TGF-β-induced EMT. Inhibiting p38 or JNK activity using specific inhibitors or gene silencing can inhibit TGF-β-induced changes in cell morphology and expression of phenotypic markers^18,28^. Similar to p38 and JNK, the ERK pathway is another non-Smad signaling pathway necessary for TGF-β-induced EMT^29^. *Liang et al*. reported that thalidomide treatment significantly inhibited TGF-β-induced p38 phosphorylation but did not have a significant effect on ERK or JNK phosphorylation^24^. An experimental *in vivo* pulmonary fibrosis rat model demonstrated that thalidomide reduced the degree of bleomycin-induced pulmonary fibrosis by down-regulating the expression of p-JNK and α-SMA^15^. Moreover, *Choe et al*. reported that the anti-fibrotic effect of thalidomide on pulmonary fibrosis might be related to its inhibition of TGF-β1-induced activation of the ERK1/2 signaling pathway^14^. In the present study, we found that TGF-β1 activated these MAPK cascades and that the TGF-β1-induced increase in the expression of mesenchymal markers, including α-SMA and vimentin, was inhibited by p38-, JNK- and ERK-specific inhibitors. This suggests that the TGF-β1-induced increase in the expression of α-SMA and vimentin is mediated by the activation of these MAPKs. We also observed that thalidomide treatment resulted in a significant reduction in the levels of phosphorylated p38, JNK and ERK in CCL-149 cells, indicating that the inhibitory effect of thalidomide on TGF-β1-induced EMT is associated with the suppression of MAPK signaling pathways.

Furthermore, several studies have suggested that the PI3K/Akt pathway is another non-Smad pathway that contributes to TGF-β1-induced EMT. Inhibition of Akt activity attenuates TGF-β1-induced EMT in kidney epithelial cells^30^. In this study, we observed that thalidomide inhibited TGF-β1-induced phosphorylation of Akt and that a specific PI3K inhibitor inhibited Akt activation and α-SMA production after TGF-β1 stimulation. This suggests that the attenuation of TGF-β1-induced EMT by thalidomide is associated with the PI3K/Akt pathway. In addition, the activation of GSK-3β, a downstream effector of Akt, was suppressed by thalidomide treatment. Previous studies have shown that inactivation of GSK-3β could lead to the stabilization of key mediators of EMT, including SNAI1 and β-catenin^31,32^. These findings lead us to hypothesize that the inhibitory effect of thalidomide on TGF-β1-induced EMT may be partly mediated by inhibition of the PI3-K/Akt/GSK-3β pathway.

In a bleomycin model of pulmonary fibrosis, approximately one third of fibroblasts are of epithelial origin^33^. EMT may also contribute to fibroblastic foci and fibrotic development in idiopathic pulmonary fibrosis (IPF)^34,35^. However, Rock *et al*. suggested that epithelial cells do not generate myofibroblasts in the context of pulmonary fibrosis^9^. These contradictory results may be due to differences in the study methods, the pulmonary fibrosis model, or the cell lines. Collectively, the role of EMT in the development of pulmonary fibrosis still needs to be confirmed in future studies. Our study provides some insight into the mechanism by which thalidomide is able to prevent EMT in a pulmonary epithelial cell line. Finally, there were three main limitations in this study. First, although we identified that both Smad-dependent and non-Smad-dependent pathways were involved in TGF-β1-induced EMT in pulmonary epithelial cell lines, we failed to determine which pathway plays the main role in this pathological process. Second, further studies are required to investigate the relationship between the Smad-dependent and non-Smad-dependent pathways during TGF-β1-induced EMT in pulmonary epithelial cells. Third, we used a fetal rat pulmonary epithelial cell line (CCL-149) instead of a human pulmonary epithelial cell line (such as A549) in this study. Thus, the different characteristics between species should be considered.

## Conclusions

The results of this study demonstrate that treatment with thalidomide inhibits TGF-β1-induced α-SMA and vimentin production and TGF-β1-induced cell morphological changes in pulmonary epithelial cells by suppressing both Smad-dependent and non-Smad-dependent pathways. Thus, thalidomide may be valuable as a therapeutic agent for the inhibition of lung fibrosis.

## Materials and Methods

### Antibodies and Reagents

TGF-β1 was purchased from R&D Systems (Minneapolis, MN, USA). TGF-β1 receptor kinase inhibitor (SB431542) was purchased from Selleck (Houston, TX, USA). PI3K/Akt inhibitor (LY294002) was purchased from Cell Signaling Technology (Beverly, MA, USA). JNK inhibitor (SP600125) and ERK inhibitor (PD098059) were purchased from Millipore Co. (Billerica, MA, USA). Thalidomide was purchased from Sigma-Aldrich (St. Louis, MO, USA). Anti-rat p38 (ab7952), p-p38 (ab4822), Smad2/3 (ab63672) and p-Smad2/3 (ab63399) were purchased from Abcam (Shanghai, China). Anti-rat ERK (BS1121) and E-Cadherin (BS1097) were purchased from Bioworld (Minneapolis, USA). Anti-rat p-ERK (#4370), AKT (#9272), p-AKT (#2965s), GSK3β (#9315) and p-GSK3β (#9336) were purchased from Cell Signaling (Beverly, MA, USA). Anti-rat JNK (sc-571), p-JNK (sc-81502) and α-SMA (sc-71626) were purchased from Santa Cruz (CA, USA). Anti-rat vimentin (3634–100) was purchased from Biovision (Mountain View, CA, USA). Anti-rat ZO-1 was purchased from Invitrogen (Carlsbad, CA, USA). Anti-β-actin and HRP-labeled secondary antibodies were purchased from Boster (Wuhan, Hubei, China). Commercial ELISA kits for matrix metalloproteinase (MMP)-2 and MMP-9 were purchased from R&D Systems (Shanghai, China). ELISA kit for collagen type IV was purchased from BlueGene Biotech (Shanghai, China).

### Cell culture and treatments

Rat lung epithelial cells (CCL-149) were purchased from Wuhan Biofavor Biotech Service CO., LTD (Wuhan, Hubei, China). Cells were cultured in DMEM/F12 supplemented with 10% fetal bovine serum (Invitrogen, San Diego, CA) and 1% streptomycin-penicillin solution. Cultures were maintained in a humidified 5% CO_2_ incubator at 37°C. After 24 hours of serum starvation, cells were maintained in growth media supplemented with 5 ng/mL TGF-β1 (dissolved in 10 mM citric acid, pH = 3.0) for 48 h to induce EMT^36^. In the majority of experiments, cells were maintained in media supplemented with TGF-β1 with or without thalidomide (1, 10, or 100 μg/mL) for 48 h to investigate the effects of thalidomide on EMT in CCL-149 epithelial cells. To investigate pathways possibly involved in the effect of thalidomide on EMT, the cells were preincubated for 1 h with SB431542 (5 μM), LY294002 (3 μM), SB203580 (20 μM), SP600125 (10 μM) and PD098059 (40 μM) before treatment with exogenous TGF-β1 with or without thalidomide. The concentrations of thalidomide and inhibitors were determined according to the results of our pilot study. Cells were harvested at the indicated time points for protein extraction and western blotting and the cell culture media was collected for ELISA analysis.

### Smad2 and Smad3 siRNA transfection

Pre-designed Smad2 (Ambion, Austin, TX) and Smad3 (Thermo, PA, USA) siRNAs were transfected into cells using siPORT™ *NeoFX*™ Transfection Agent (Ambion) at a concentration of 20 nM. The siRNA-transfection reagent complexes were prepared as described by the manufacturer. Cells were treated with or without 5 ng/mL TGF-β1 in the presence or absence of different concentrations of thalidomide for 48 h. The efficiency of siRNA gene silencing was evaluated with western blots for Smad2 and Smad3. The effect of siRNA transfection on TGF-β1-induced α-SMA production was analyzed by western blotting as described below.

### Western blot assay

Cells were lysed in RIPA (P0013B, Beyotime Biotechnology, Shanghai, China) and protein concentrations were determined using the BCA protein assay kit (P0010, Beyotime Biotechnology, Shanghai, China). Equal amounts of proteins (50 μg) were resolved by 10% SDS-PAGE and the proteins were transferred to Hybond ECL membranes (Amersham, Buckinghamshire, UK). The membranes were incubated with primary antibodies at 4°C overnight. The primary antibodies used included anti-rat p38 (1:300), phosphorylated p38 (1:1000), vimentin (1:300), ERK (1:600), phosphorylated ERK (1:1200), Smad2/3 (1:300), phosphorylated Smad2/3 (1:600), E-Cadherin (1:600), α-SMA (1:300), JNK (1:300), phosphorylated JNK (1:300), AKT (1:800), phosphorylated AKT (1:800), GSK3β (1:1000), phosphorylated GSK3β (1:1000) and ZO-1 (1:250). After washing with TBST, the membranes were probed with HRP-labeled secondary antibodies. The membranes were visualized using an enhanced chemiluminescence system (Kodak, Rochester, NY, USA). β-Actin was used as a loading control. To normalize the intensities of different membranes, standard samples were performed on every blot. Then, the densitometric data of Western blots of the three independent experiments were combined.

### ELISA

As described above, the cell culture media was collected for the detection of MMP-2, MMP-9 and collagen type IV secreted from CCL-149 cells in response to pretreatment with TGF-β1 and thalidomide. Culture media was centrifuged at 3000 rpm for 20 min. Quantification of MMP-2, MMP-9 and collagen type IV was performed using commercial ELISA kits according to the manufacturer’s instructions.

### Immunofluorescence staining

Immunofluorescence staining was performed as previously described^37^. Briefly, cells cultured on coverslips were washed thrice with PBS and fixed with paraformaldehyde (4%) for 15 min. Following three washes with PBS, the cells were blocked with 0.1% Triton X-100 and 2% normal serum for 30 min at room temperature (RT) and then incubated with specific primary antibodies, including those against α-SMA (BM0002, Boster, Wuhan, China) and E-Cadherin (sc-7870, Santa Cruz, CA, USA), at 4°C overnight. The cells were then stained with the appropriate secondary antibodies (Boster, Wuhan, China). The cells were also stained with 4,6-diamidino-2-phenylindole (DAPI) to visualize the nuclei and were washed with PBST four times for 5 min each time. The slides were viewed with an epi-fluorescence microscope (Nikon Eclipse 80i, Nikon, Tokyo, Japan) equipped with a digital camera (DS-Ri1, Nikon, Tokyo, Japan).

### Statistical analysis

Data are expressed as the means ± SD. Data were analyzed using the statistical analysis software SPSS version 20.0 (SPSS Inc., Chicago, IL, USA). Comparisons of several means were performed using one-way and repeated measure two-way analysis of variance followed by the Tukey-Kramer test to identify significant differences between groups. All *P* values were two-tailed, and a *P* value of less than 0.05 was considered significant.

## References

[1] King TE, Pardo A, Selman M (2011). Idiopathic pulmonary fibrosis. Lancet.

[2] Nawshad A, Lagamba D, Polad A, Hay ED (2005). Transforming growth factor-beta signaling during epithelial-mesenchymal transformation: implications for embryogenesis and tumor metastasis. Cells Tissues Organs.

[3] Willis BC (2005). Induction of epithelial-mesenchymal transition in alveolar epithelial cells by transforming growth factor-beta1: potential role in idiopathic pulmonary fibrosis. Am J Pathol.

[4] Chen, H. H., Zhou, X. L., Shi, Y. L. & Yang, J. Roles of p38 MAPK and JNK in TGF-beta1-induced human alveolar epithelial to mesenchymal transition. *Arch Med Res* **44**, 93–98 (2013). 10.1016/j.arcmed.2013.01.004 23376055

[5] Willis BC, Borok Z (2007). TGF-beta-induced EMT: mechanisms and implications for fibrotic lung disease. Am J Physiol Lung Cell Mol Physiol.

[6] Xi Y (2014). Inhibition of epithelial-to-mesenchymal transition and pulmonary fibrosis by methacycline. Am J Respir Cell Mol Biol.

[7] Felton VM, Borok Z, Willis BC (2009). N-acetylcysteine inhibits alveolar epithelial-mesenchymal transition. Am J Physiol Lung Cell Mol Physiol.

[8] Chu AS (2011). Lineage tracing demonstrates no evidence of cholangiocyte epithelial-to-mesenchymal transition in murine models of hepatic fibrosis. Hepatology.

[9] Rock JR (2011). Multiple stromal populations contribute to pulmonary fibrosis without evidence for epithelial to mesenchymal transition. Proc Natl Acad Sci USA.

[10] O'Beirne SL (2015). CXCL9 Regulates TGF-beta1-Induced Epithelial to Mesenchymal Transition in Human Alveolar Epithelial Cells. J Immunol.

[11] Wang, Y. *et al*. Maresin1 Inhibits Epithelial-to-Mesenchymal Transition *in vitro* and Attenuates Bleomycin Induced Lung Fibrosis *in vivo*. *Shock* (2015). 10.1097/SHK.0000000000000446 26196843

[12] Tabata C (2007). Thalidomide prevents bleomycin-induced pulmonary fibrosis in mice. J Immunol.

[13] Amirshahrokhi K (2013). Anti-inflammatory effect of thalidomide in paraquat-induced pulmonary injury in mice. Int Immunopharmacol.

[14] Choe JY (2010). Anti-fibrotic effect of thalidomide through inhibiting TGF-beta-induced ERK1/2 pathways in bleomycin-induced lung fibrosis in mice. Inflamm Res.

[15] Liu X (2014). Function of the transforming growth factor-beta1/c-Jun N-terminal kinase signaling pathway in the action of thalidomide on a rat model of pulmonary fibrosis. Exp Ther Med.

[16] Liang CJ (2013). Thalidomide inhibits fibronectin production in TGF-beta1-treated normal and keloid fibroblasts via inhibition of the p38/Smad3 pathway. Biochem Pharmacol.

[17] Zhou B (2012). Troglitazone attenuates TGF-beta1-induced EMT in alveolar epithelial cells via a PPARgamma-independent mechanism. PLoS One.

[18] Kolosova I, Nethery D, Kern JA (2011). Role of Smad2/3 and p38 MAP kinase in TGF-beta1-induced epithelial-mesenchymal transition of pulmonary epithelial cells. J Cell Physiol.

[19] Jia L (2015). Sorafenib ameliorates renal fibrosis through inhibition of TGF-beta-induced epithelial-mesenchymal transition. PLoS One.

[20] Bi WR, Yang CQ, Shi Q (2012). Transforming growth factor-beta1 induced epithelial-mesenchymal transition in hepatic fibrosis. Hepatogastroenterology.

[21] Massague J, Wotton D (2000). Transcriptional control by the TGF-beta/Smad signaling system. EMBO J.

[22] Saika S (2004). Smad3 is required for dedifferentiation of retinal pigment epithelium following retinal detachment in mice. Lab Invest.

[23] Phanish MK, Wahab NA, Colville-Nash P, Hendry BM, Dockrell ME (2006). The differential role of Smad2 and Smad3 in the regulation of pro-fibrotic TGFbeta1 responses in human proximal-tubule epithelial cells. Biochem J.

[24] Liang CJ (2013). Thalidomide inhibits fibronectin production in TGF-beta1-treated normal and keloid fibroblasts via inhibition of the p38/Smad3 pathway. Biochem Pharmacol.

[25] Kobayashi T (2006). Smad3 mediates TGF-beta1-induced collagen gel contraction by human lung fibroblasts. Biochem Biophys Res Commun.

[26] Yu L, Hébert MC, Zhang YE (2002). TGF-beta receptor-activated p38 MAP kinase mediates Smad-independent TGF-beta responses. EMBO J.

[27] Engel ME, McDonnell MA, Law BK, Moses HL (1999). Interdependent SMAD and JNK signaling in transforming growth factor-beta-mediated transcription. J Biol Chem.

[28] Liu Q (2012). A crosstalk between the Smad and JNK signaling in the TGF-beta-induced epithelial-mesenchymal transition in rat peritoneal mesothelial cells. PloS One.

[29] Davies M (2005). Induction of an epithelial to mesenchymal transition in human immortal and malignant keratinocytes by TGF-beta1 involves MAPK, Smad and AP-1 signalling pathways. J Cell Biochem.

[30] Kattla JJ, Carew RM, Heljic M, Godson C, Brazil DP (2008). Protein kinase B/Akt activity is involved in renal TGF-beta1-driven epithelial-mesenchymal transition *in vitro* and *in vivo*. Am J Physiol Renal Physiol.

[31] Zhou BP (2004). Dual regulation of Snail by GSK-3beta-mediated phosphorylation in control of epithelial-mesenchymal transition. Nat Cell Biol.

[32] Bowley E, O'Gorman DB, Gan BS (2007). Beta-catenin signaling in fibroproliferative disease. J Surg Res.

[33] Tanjore H (2009). Contribution of epithelial-derived fibroblasts to bleomycin-induced lung fibrosis. Am J Respir Crit Care Med.

[34] Willis BC (2005). Induction of epithelial-mesenchymal transition in alveolar epithelial cells by transforming growth factor-beta1: potential role in idiopathic pulmonary fibrosis. Am J Pathol.

[35] Willis BC, duBois RM, Borok Z (2006). Epithelial origin of myofibroblasts during fibrosis in the lung. Proc Am Thorac Soc.

[36] Kasai H, Allen JT, Mason RM, Kamimura T, Zhang Z (2005). TGF-beta1 induces human alveolar epithelial to mesenchymal cell transition (EMT). Respir Res.

[37] Xiong M (2012). The miR-200 family regulates TGF-beta1-induced renal tubular epithelial to mesenchymal transition through Smad pathway by targeting ZEB1 and ZEB2 expression. Am J Physiol Renal Physiol.

